# Radiation dosimetry and fasting-dependent hepatobiliary clearance of the VAChT-specific PET radioligand ^18^F-VAT in humans

**DOI:** 10.1186/s13550-025-01273-z

**Published:** 2025-07-07

**Authors:** Scott A. Norris, Noah L. Goldman, Mahdjoub Hamdi, Stephen M. Moerlein, Richard Laforest, Morvarid Karimi, Joel S. Perlmutter, Zhude Tu

**Affiliations:** 1 Department of Neurology, Washington University School of Medicine, 660 South Euclid Ave, CB 8111, St. Louis, MO 63110 USA; 2 Mallinckrodt Institute of Radiology, Washington University School of Medicine, St. Louis, MO 63110 USA; 3 Department of Neuroscience, Washington University School of Medicine, St. Louis, MO 63110 USA; 4 Program in Physical Therapy, Washington University School of Medicine, St. Louis, MO 63110 USA; 5 Program in Occupational Therapy, Washington University School of Medicine, St. Louis, MO 63110 USA

**Keywords:** PET, VAChT, Radiation dosimetry, Vesicular acetylcholine transporter

## Abstract

**Background:**

The vesicular acetylcholine transporter ligand (-)-(1-((2R,3R)-8-(2-[(18)F]fluoro-ethoxy)-3-hydroxy-1,2,3,4-tetrahydronaphthalen-2-yl)piperidin-4-yl)(4-fluorophenyl)-methanone (^18^F -VAT) enables positron emission tomography PET quantification of cholinergic dysfunction in neurologic and psychiatric disorders. Determining its bio-distribution and dose exposure in humans is essential for clinical implementation, particularly given hepatobiliary clearance observed in pre-clinical models. Based on pre-clinical data, eight healthy subjects (4 males, 4 females) received 385–533 MBq ^18^F-VAT immediately followed by three sequential whole-body PET/CT scans. PET data were collected under three different fasting conditions relative to administration of Ensure®Plus oral supplement and PET image acquisition: (1) complete fasting (n = 3), (2) oral partial fasting (n = 3), or (3) non-fasting (n = 2). We defined volumes of interest (VOIs), and generated organ time-activity curves (TACs). Organ radiation dosimetry was calculated using OLINDA/EXM v2.2 software.

**Results:**

There were no adverse events after ^18^F-VAT dosing. Radioactivity accumulated predominantly in the brain, hepatobiliary system, small intestine, bone, and urinary bladder. Across all fasting states, organ dosimetry revealed gallbladder as the critical organ (201.0 μSv/MBq) followed by liver (64.3 μSv/MBq), with a gender averaged effective dose of 17.5 ± 2.1 μSv/MBq (15.7 and 19.4 μSv/MBq for males and females, respectively.) Mean gallbladder time integrated activity significantly differed across non-fasting (36.6 MBq*h, 155.5 µSv/MBq), partial fasting (21.8 MBq*h, 107.6 µSv/MBq) and fasting PET acquisition (74.1 MBq*h, 270.5 µSv/MBq) (Kruskal–Wallis H 6.5, p = 0.04).

**Conclusions:**

Human bio-distribution data showed high retention of ^18^F-VAT in the gallbladder and liver, where rat dosimetry studies do not accurately predict a safety profile given lack of gallbladder. Human dosimetry data appear different from fasting non-human primate data, indicating that up to 249 MBq (6.7 mCi) of ^18^F-VAT can be administered without exceeding a maximum dose to the gallbladder of 50 mSv (5 rem) without consideration of fasting state. Oral supplementation, administered just before and especially 90 min after ^18^F-VAT administration, accelerates gallbladder clearance. This reduces critical organ radiation exposure, allowing an administered dose of ^18^F-VAT to 465 MBq (12.6 mCi) in the optimal partial fasting state without exceeding a gallbladder dose of 50 mSv (5 rem).

## Background

Acetylcholine, a key neurotransmitter in the central nervous system, is synthesized in nerve terminals by choline acetyltransferase (ChAT) and transported into synaptic vesicles via the vesicular acetylcholine transporter (VAChT) protein [1]. Both ChAT and VAChT are highly expressed in cholinergic neurons [2] that are implicated in various neurologic and psychiatric disorders [3–5]. Imaging techniques like single-photon emission computed tomography (SPECT) [6] and positron emission tomography (PET) [7, 8] using cholinergic radiotracers can quantify the regional distribution of cholinergic neurons in the human brain. The PET ligand vesicular acetylcholine transporter ((-)-(1-(-8-(2-fluoroethoxy)-3-hydroxy-1,2,3,4-tetrahydronaphthalen-2-yl)piperidin-4-yl)(4-fluorophenyl)-methanone) (^18^F-VAT), a high-affinity VAChT ligand, shows substantial promise for assessing VAChT density and function [9–13] (Jin et al., 2018) [14].

Accurate assessment of ^18^F-VAT radiation dosimetry in humans is important for safe and effective use in research and potential clinical settings. Prior studies have used rat bio-distribution data to predict human dosimetry [12]. However, this may be inadequate because ^18^F-VAT uptake is highest in the hepatobiliary system, and rats lack a gallbladder, which is crucial for hepatobiliary tracer clearance in humans. Other studies obtained dosimetry estimation of ^18^F-VAT in fasting, anesthetized non-human primates, revealing high localization in the liver consistent with hepatobiliary clearance [15]. While prior non-human primate studies may better predict human dosimetry than rat studies, no studies have directly addressed the safety profile or effects of fasting state on hepatobiliary clearance of the ^18^F-VAT radiotracer in humans. The primary objective of this study is to determine the maximum safe dose of ^18^F-VAT in humans; specifically accounting for the potential role that a fasting state may have on hepatobiliary clearance of this tracer. These data may allow clinical investigators to optimize ^18^F-VAT PET imaging protocols in humans, following federal regulations for dosimetry and supporting translational research. Such knowledge may specifically facilitate longitudinal studies that explore the role of VAChT in neurodegenerative disorders, and potentially pave the way for development of novel diagnostic tools and therapeutic interventions.

## Methods

### Study design and participants

The Washington University Institutional Review Board and Radioactive Drug Research Committee approved this study (#201501149) performed under FDA Investigational New Drug Application #125627. All participants provided written informed consent prior to inclusion. The dosimetry cohort included eight healthy volunteer participants (4 female, 4 male, age 23–67) who underwent whole-body PET/CT imaging. Inclusion criteria were any sex or race, age greater than 18 years, and ability to provide written informed consent. Criteria for exclusion consisted of any diagnosed neurological disease, active pregnancy (urine pregnancy test to exclude), actively breastfeeding, current exposure to clinical radiation therapy or a research PET scan within 12 months, any exposure to drugs that block dopamine receptors, CNS active medications that affect dopaminergic or cholinergic systems three days prior to study, history of addiction, active depression, psychotic illness, cognitive impairment (Montreal Cognitive Assessment < 26), history of unstable general medical condition, and weight over 300 pounds.

### Radiopharmaceutical preparation

^18^F-VAT was prepared under cGMP conditions done in the Mallinckrodt Institute of Radiology Cyclotron Facility and Nuclear Pharmacy at Washington University School of Medicine as previously described [14, 16]. In brief, [^18^F]fluoroethyl tosylate was produced using an Eckert and Ziegler Modular-Lab system [12, 17, 18] and [^18^F]VAT was synthesized with the GE TRACERlab FX-N module as [^18^F]fluoroethyl tosylate reacted with the corresponding enantiomerically pure phenol precursor. The drug substance was purified by reverse phase HPLC, and the parenteral product was sterilized by membrane filtration and reformulated in 10% ethanol in 0.9% Sodium Chloride Injection, USP. The sterile, apyrogenic product met a battery of pre-release quality control tests, including radiochemical purity > 99%, injected mass of VAT < 5 μg, and specific activity > 111 GBq/μmol.

### PET/CT data acquisition

The height and weight of each participant was recorded prior to scan. Given prior pre-clinical studies indicating hepatobiliary clearance of ^18^F-VAT, we designed the study to include ^18^F-VAT administration and multiple whole-body PET acquisitions (i.e., segment) in three different fasting states as described here: (1) “complete fasting”: after overnight fasting, ^18^F-VAT administration and three whole-body PET segments, (2) “partial fasting”: after overnight fasting, ^18^F-VAT administration plus one PET whole-body segment followed by Ensure® Plus oral supplementation and two PET segments, and (3) “non-fasting” after overnight fasting, Ensure® Plus oral supplementation immediately prior to ^18^F-VAT administration followed by three whole-body PET segments. For the complete fasting state, participants #1, #2, #4 and #5 were instructed to fast at midnight prior to the study day through the end of the PET scan. One participant (#2) consumed a small breakfast consisting of milk and cereal due to discomfort, approximately 2 h prior to PET scan- this participant is thus considered “partial fasting” henceforth. For the intended partial fasting state, participants #3 and #7 fasted at midnight prior to the study day, until they consumed oral Ensure® Plus between PET segments one and two (see below). In the non-fasting state, participants #4 and #8 fasted at midnight prior to study day, then consumed oral Ensure® Plus in the 30 min prior to ^18^F-VAT administration and all subsequent PET scans.

**Fig. 1:**
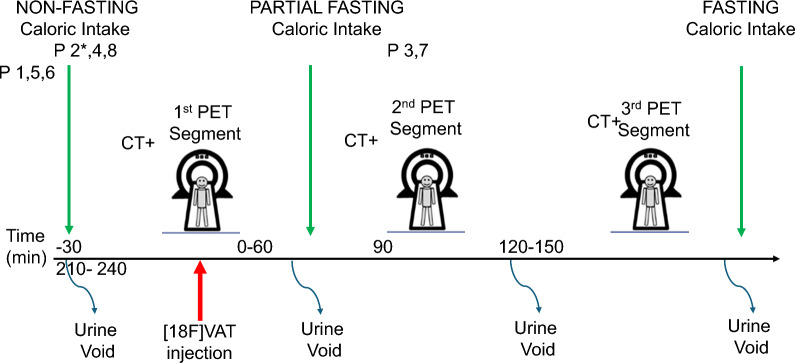
Timeline of study day events around PET data acquisition

All participants voided their bladder before^18^F-VAT administration and the PET imaging session. A 22-gauge plastic catheter was inserted into the right antecubital vein to permit radiotracer administration. We used surgical tape to secure the head position of the awake participant. PET data were obtained using a Siemens Vision Biograph PET/CT (Siemens Healthineers, Knoxville TN, USA) with participants scanned in the supine position at rest for three independent whole-body dynamic PET segments. Prior to each individual PET segment, a topogram was obtained (35mAs, 120 kV) followed by low-dose spiral CT scan for attenuation correction (50mAs effective, 120 kV) covering from the top of the skull through the upper thighs. Immediately after the first attenuation CT scan, three dynamic PET segments were similarly obtained from the top of skull through the upper thighs beginning at initiation of a 20 s bolus IV injection of ^18^F-VAT (389–533 MBq, 10.5–14.4 mCi) followed by immediate saline flush of the intravenous catheter. The first PET segment (sequential whole body [WB] scans 1 to 17) began with the start of the bolus injection and lasted for approximately 60 min (time per whole body scan was 60 s [WB 1–5], 120 s [WB 6–10], and 360 s [WB 11–17], respectively). Participants #3 and #7, in the partial fasting group, consumed oral Ensure® Plus in the 30 min prior to the second PET segment. The second PET segment (WB 18–22) started 2 h post-injection, collecting 360 s per whole body scan. The third PET segment (WB 23–25) started 3.5 h post-injection, collecting 600 s per whole body scan. All participants exited the PET scanner between segments and were offered an opportunity to void their bladder. This procedure aimed to enhance comfort and reduce the risk of disrupting subsequent PET sequence due to a sudden urge to void. All participants were allowed to eat immediately following completion of the third and final PET segment. Figure 1 represents the study sequence across participants.

### PET/CT image reconstruction

For the purpose of image reconstruction, the low-dose CT performed before each PET segment provided attenuation correction and anatomical information. Whole-body PET scan images were reconstructed using the ordered subset expectation maximization (OSEM) algorithm with time of flight (TOF) and point spread function (PSF) resolution modeling (4 iterations and 5 subsets) with no post-reconstruction filtering. The reconstruction resolution was 1.65 × 1.65 × 3-mm and included corrections for scatter, randoms, and attenuation based on CT. We inspected images for evidence of motion, and alignment was manually corrected, if needed.

### VOIs

Three-dimensional volumes of interest (VOIs) were manually traced using MIM® software (version 6.9.3) over source organs with measurable activity, defined as signal increase above background levels sufficient to allow visual recognition: brain, lungs, heart (wall and left ventricle), liver, gallbladder, spleen, small intestine, kidneys, urinary bladder, and the lumbar vertebrae (L1–L3). Organs with well-defined boundaries were contoured in their entirety (e.g., liver and kidneys). Organs with complex boundaries (e.g., small bowel) were sampled and activity scaled to the estimated mass of the organ. To address intra-scan motion, we analyzed the tracer distribution within identified VOIs across all acquired frames for each PET segment. If the VOIs did not accurately estimate tracer uptake in the targeted organ for all frames, a new VOI was delineated to better represent the biodistribution within the respective organ for affected frames.

### Radiation dosimetry

Time-activity curves (TAC) were obtained for each source organ by calculating the total activity in the organ-specific VOIs and expressing them as the percentage of the total injected activity. Given the time-course of activity in a volume of tissue, residence times, or normalized cumulated activity, were then calculated with the trapezoid method, except for the bladder as described below. We elected to apply the trapezoid method to avoid overfitting data in context of unevenly and undersampled sampled data between PET segments, and as we observe narrow peaks in time activity curves for some VOIs. Specifically, given an activity A_0_ (injected dose) applied to a participant, and measured (not decay corrected) A(t) in an organ, the residence time τ is calculated by:

1 $$\uptau =\frac{{\int }_{0}^{\infty }A\left(t\right)dt}{{A}_{0}}$$

Calculated residence time values were entered into the OLINDA/EXM 2.2 software package [19] for F-18 using the adult human male and female anthropomorphic models as done in prior work [20–23] to generate the organ radiation dose.

For the bladder, the cumulative activity was fitted by an uptake function of the form:

2 $$\text{F}(\text{t}) =\text{ A}0*(1-1{\text{e}}^{\left(-\text{A}1\bullet \text{t}\right)})$$

where A0 and A1 were fitting parameters representing the filling fraction and filling constant. For dosimetry modeling, the urinary bladder was assumed to be voided every 2 h, consistent with the study protocol in which participants were instructed to void prior to each PET session. This assumption was implemented in the OLINDA bladder model to best reflect the conditions under which activity accumulation was observed and measured.

The time-integrated activity is calculated by:

3 $$\ A = \mathop \smallint \limits_{0}^{\infty } A\left( t \right)dt$$

## Results

### Participant demographics and administered dose

Table 1 displays demographic and radiopharmaceutical dosing information for the four ^18^F-VAT participants.

**Table 1:** Participant demographics

Participant	Sex	Handedness	Age (yr)	Height (m)	Weight (kg)	PET fasting group	Injected dose (MBq)
1	Female	Right	64	1.63	88	Complete fasting	518
2	Female	Right	64	1.63	103.3	Partial fasting*	525
3	Female	Right	38	1.65	58.2	Partial fasting	389
4	Female	Right	28	1.65	96.2	Non-fasting	507
5	Male	Right	27	1.75	70.3	Complete fasting	533
6	Male	Right	23	1.91	94.8	Complete fasting	533
7	Male	Right	50	1.78	72.6	Partial fasting	519
8	Male	Right	67	1.63	72.6	Non-fasting	514

*Despite intent for complete fasting, this participant consumed a small breakfast of milk and cereal approximately 2-h before ^18^F-VAT administration and PET acquisition due to discomfort

### Adverse events

There were no adverse or clinically detectable effects observed or reported following ^18^F-VAT administration in any participant. No participant reported injection site reactions or any other symptoms during the procedure, throughout the duration of the 4-h scan, or in telephone follow-up one day after the PET scan.

### Human biodistribution

**Fig. 2:**
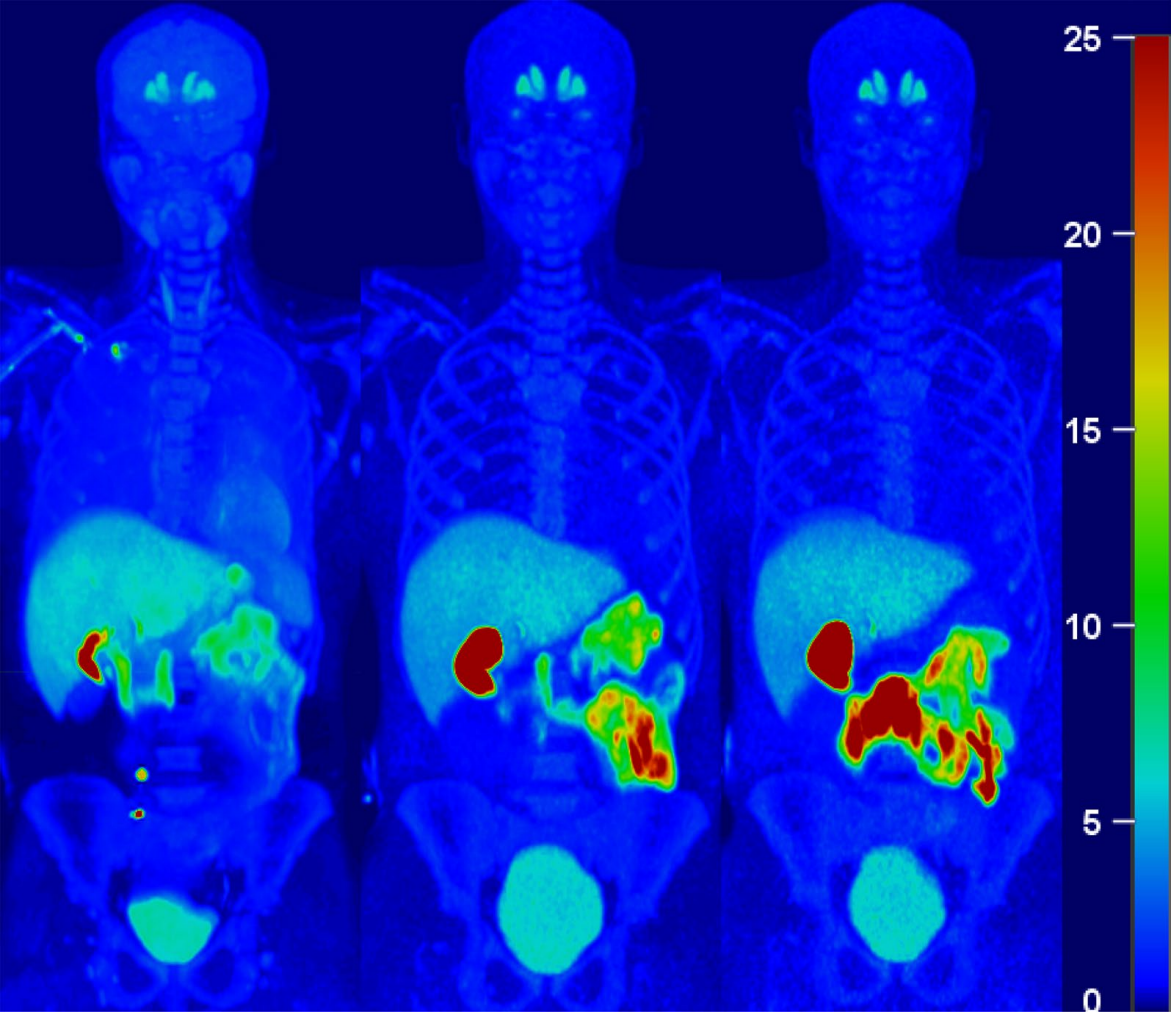
Participant #6 maximum intensity coronal projection (MIP) of all PET frames in the **A** first, **B** second, and **C** third PET segments with decay-corrected standard uptake values (SUV) demonstrating high uptake in the muscle, liver, gallbladder, small intestine, urinary bladder, as well as brain

Figure 2 shows the whole-body PET data averaged for each of three segments in participant 4 fused onto the corresponding CT scan. This representative image demonstrates significant radioligand uptake in the muscle, liver, gallbladder, small intestine, urinary bladder, as well as brain (specifically in the caudate and putamen). High activity was observed in the liver at early time points whereas the gallbladder and small intestine had higher activity at later time points, compatible with hepatobiliary excretion. Minimal activity was observed in the kidneys despite accumulation in the urinary bladder.

### Time-activity curves

**Fig. 3:**
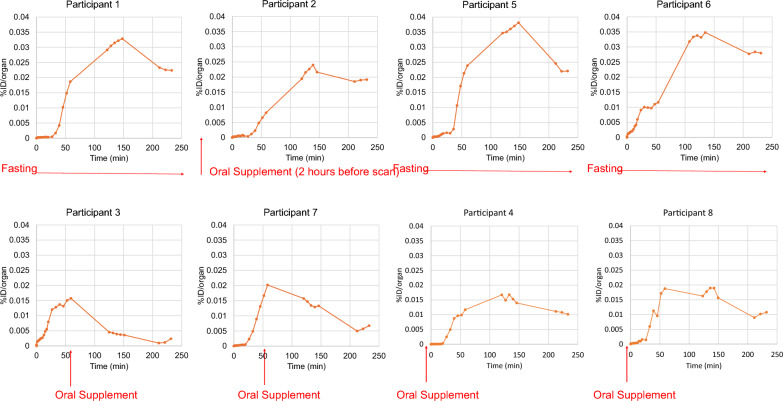
Non-decay-corrected organ time-activity curves for participant 6. %ID = percentage injected dose

We calculated TACs in organs with measurable activity from the PET images. Figure 3 shows TACs compiled for a single representative non-fasting participant (#4).

We observed a rapid accumulation and slow clearance of activity in muscle, lung, and brain, with initial fractions at ~ 18%, ~ 15%, and 4% of the injected dose, respectively. The liver and gallbladder reflected a delayed accumulation of ~ 25% and ~ 5%, of injected dose, respectively. For the liver, peak accumulation occurred at approximately 35 min after injection, followed by a slow clearance.

### Effect of feeding on gall bladder accumulation

**Fig. 4:**
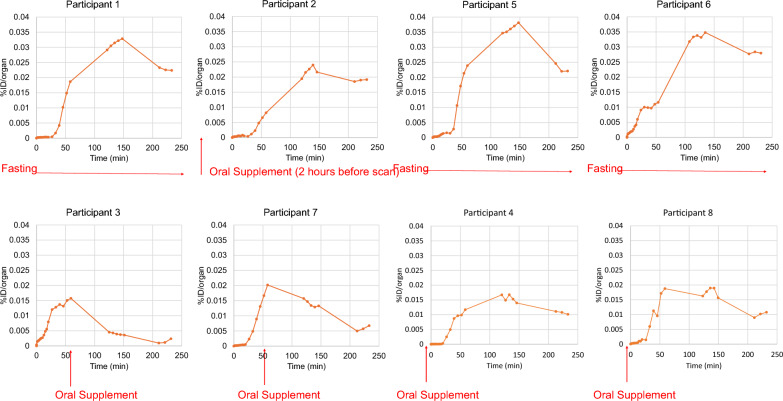
Non-decay corrected gallbladder TACS for individual participants. Red text describes fasting versus non-fasting states, where the time of oral supplementation is indicated relative to PET data acquisition. Note trends of increasing TAC across PET segments in the fasting state (participants 1,5,6), reduction in TAC following oral supplementation when administered between PET segments 1&2 (participants 3,7) and reduced overall TAC when oral supplementation is administered prior to PET acquisition (participants 4 and 8). Participant #2 was intended to fast but had a small mean 2 h prior to PET acquisition

As shown in Fig. 4, for subjects in the fasting group (1. 5. 6), the gallbladder accumulated radioactivity throughout the course of PET image acquisition, demonstrating a 20% *increase* of combined TAC data of segments 2 and 3 compared to segment 1. This accumulation effect was reversed in the two participants in the partial fasting group (3 and 7), in which there was a 59% *decrease* of the integrated TAC of combined segments 2 and 3 compared to segment 1. For the non-fasting subjects (4 and 8), there was no substantial accumulation of radioactivity in the gallbladder, and the TAC for segments 1–3 were low and not substantially different.

### Time integrated activity

Time integrated activity was calculated for all organs (Tables 2, 3, 4, 5). The organ with the greatest time integrated activity time was the muscle tissue followed by liver, bone marrow and gallbladder, while the smallest were the kidneys. The low standard deviation on the time integrated activity for all organs suggests good reproducibility. Mean gallbladder time integrated activity differed significantly between the fasting group (74.1 MBq*h) and the combined non-fasting (36.6 MBq*h), and partial fasting (21.8 MBq*h) groups (Kruskal–Wallis H 6.5, p = 0.03).

**Table 2:** Time-integrated activity of female participants in MBq

	Participant number			
Site	1*	2^†§^	3^†^	4^‡^
Brain	56.9	38.7	31.4	24.6
Lungs	13.6	17.9	10.3	26
Heart	22.3	13.5	9.2	5.3
Blood LV	9.9	15.6	4.8	4
Liver	155.6	99.3	214.2	186
Gallbladder	72.7	55.1	12.3	33.5
Spleen	18.2	4.1	4.7	5.7
Small Intestine	37.2	36	71.4	36.8
Kidneys	9.3	8.3	6.9	7.5
Bladder	6.7	13.2	28.1	38.8
Bone Marrow	6.7	37.9	58	46.9
Muscle	273.4	226.9	125	147.1
Remainder	665.8	802.2	436.4	681.4

*Complete fasting

^†^Partial fasting

^‡^Non-fasting

^§^intent was complete fasting, but participant elected to eat 2-h prior to 18F-VAT injection/PET acquisition due to general discomfort

**Table 3:** Time-integrated activity of male participants in MBq

	Participant number			
Site	5*	6*	7^†^	8^‡^
Brain	37.3	35.9	33.8	38.3
Lungs	29.3	12.7	15.2	26.5
Heart	12.2	8.7	15.8	10.5
Blood LV	13	8.2	4.5	8.8
Liver	183.6	158.4	300.9	232.5
Gallbladder	81.3	87.4	31.4	39.8
Spleen	7	5.8	6.7	8.4
Small Intestine	45.2	33.1	58.4	79.1
Kidneys	10.4	7.6	6.5	5.9
Bladder	21.2	11.5	37.5	29.7
Bone Marrow	48.9	39.3	47.4	74.5
Muscle	279.6	224.8	446.1	453.1
Remainder	616.5	757.8	352.2	337.4

*Complete fasting

^†^Partial fasting

^‡^Non-fasting

**Table 4:** Time-integrated activity in MBq averaged across all participants and non-fasting group

Site	Mean all participants	SD	Mean non-fasting	SD
Brain	37.1	9.3	31.4	9.7
Lungs	18.9	7.3	26.2	0.3
Heart	12.2	5.2	7.9	3.6
Blood LV	8.6	4.2	6.4	3.4
Liver	191.3	59.9	209.2	32.9
Gallbladder	51.7	26.8	36.6	4.5
Spleen	7.6	4.5	7.1	1.9
Small Intestine	49.6	17.8	57.9	29.9
Kidneys	7.8	1.5	6.7	1.1
Bladder	23.3	12.1	34.3	6.4
Bone Marrow	45	19.3	60.7	19.5
Muscle	272	122.3	300.1	216.4
Remainder	581.2	181.8	509.4	243.2

**Table 5:** Time-integrated activity in MBq averaged across partial-fasting and fasting group

Site	Mean partial-fasting	SD	Mean fasting	SD
Brain	32.6	1.7	42.2	9.9
Lungs	12.8	3.5	18.4	7.6
Heart	12.5	4.6	14.2	5.8
Blood LV	4.7	0.2	11.7	3.3
Liver	257.5	61.3	149.2	35.6
Gallbladder	21.8	13.5	74.1	14
Spleen	5.7	1.4	8.8	6.4
Small Intestine	64.9	9.2	37.9	5.2
Kidneys	6.7	0.3	8.9	1.2
Bladder	32.8	6.6	13.2	6
Bone Marrow	52.7	7.5	33.2	18.3
Muscle	285.5	227.1	251.2	29.4
Remainder	394.3	59.6	710.6	84.6

### Dosimetry

Dosimetry data for individual participants, averaged across all participants, males, females, and the two non-fasting participants are presented in Tables 6, 7 and 8. These data reflect effective dose for human adults. The dose-limiting organ was the gallbladder with a mean dose across all participants of 201.0 ± 80.4 µSv/MBq followed by the liver at 64.3 ± 18.4 µSv/MBq. The critical organ mean dose was similar across males (213.0 µSv/MBq) and females (189.0 µSv/MBq). The mean effective dose was 17.5 ± 2.1 µSv/MBq (15.7 µSv/MBq for males and 19.4 µSv/MBq for females). In this most conservative calculation, an injected dose of 249 MBq (6.7 mCi) would yield a total effective dose of 4.36 mSv (0.436 rem) with the critical organ, the gallbladder, receiving the maximum allowed exposure of 50 mSv (5 rem). A peri-procedural oral caloric bolus between PET segments 1 and 2 reduced the overall radiation of the gallbladder to 107.6 µSv/MBq (398.1 mrem/mCi) averaged across two participants of different genders. A pre-procedural oral caloric bolus prior to PET session reduced the overall radiation of the gallbladder to 155.5 µSv/MBq (575.4 mrem/mCi) averaged across two participants of different genders. In this situation, up to 465 MBq (12.6 mCi) of [^18^F]VAT can be administered to human participants without exceeding a maximum dose to the gallbladder of 50 mSv (5 rem).

**Table 6:** Radiation dosimetry (μSv/MBq) across all participants

	Participant number							
Site	Female	Female*	Female^†^	Female^‡^	Male	Male	Male^†^	Male^‡^
	1	2	3	4	5	6	7	8
Adrenals	27.9	21.4	27.5	25.1	21.1	20.1	23.5	22
Brain	22.4	16.2	17.1	12.1	13.5	13.1	12.4	14.1
Breasts	10.6	10.9	9.81	10.6				
Esophagus	15.9	14.9	16.3	15.6	13.2	12.6	13.2	13
Eyes	11.5	11.5	9.72	10.3	8.5	9.01	7.51	8.05
Gallbladder Wall	292	221	86.1	157	276	293	129	154
Left colon	19.2	18.6	17.7	17.3	14.9	14.8	14.4	15.7
Small intestine	38.1	36.9	71	39	34.4	29.1	40.7	51.5
Stomach wall	17.3	16.2	16.6	16.5	14.1	13.8	13.9	13.9
right colon	19.8	19	18.9	18.6	19.8	20.1	17.5	18
Rectum	14.2	16.2	14.6	15.6	12	12.4	11.8	12.3
Heart wall	42.9	34.2	28.7	19.3	26.1	20	27.4	23.7
Kidneys	27	22.8	27	24.3	21.8	18.3	19	18
Liver	57.9	39.3	96.1	70.9	54.5	48.4	81.5	66
Lungs	14.3	14.9	15	18.8	15.5	10.8	12.7	15.3
Ovaries	15.1	16.3	15.3	15.7				
Pancreas	23.8	21.2	25.9	23.4	17.1	16.7	17.7	18
Prostate					11.6	12	11.6	11.4
Salivary Glands	11.8	12.1	10	11	9.75	10.3	8.62	9.18
Red marrow	22.4	21.2	24.8	21	16.2	15.1	15.8	19.6
Osteogenic cells	16.5	16.2	16.8	15.6	13.7	13.4	12.9	15.3
Spleen	57	19.8	26.1	25.5	22.8	19.9	22.2	26.5
Testes					8.77	9.43	7.94	8.13
Thymus	14.2	14.5	12.6	13.6	11.7	11.2	10.7	11
Thyroid	11.4	12.1	10.2	11.5	10.1	10.3	8.96	9.48
Urinary Bladder wall	13.6	25.9	27.5	29.1	17.8	15.2	32.9	21
Uterus	15	16.9	16.4	16.4				
Total body	15.2	14.8	15.2	15	11.4	11.6	11	11.3
Effective dose	19.9	18.3	20	19.3	16	15	15.7	15.9

*Participant elected to eat 1 h prior to the first PET scan due to general discomfort while fasting

†Participant received PO Ensure®Plus 8-oz bolus during the PET session (between scan 1 and 2)

‡Participant received PO Ensure®Plus 8-oz bolus prior to the PET session

**Table 7:** Radiation dosimetry (μSv/MBq) across gender

Site			Average all participants (± SD)			
	Gender Mean	SD	Male	SD	Female	SD
Adrenals	23.6	3	21.7	1.4	25.5	3
Brain	15.1	3.4	13.3	0.7	17	4.2
Breasts					10.5	0.5
Esophagus	14.3	1.5	13	0.3	15.7	0.6
Eyes	9.5	1.5	8.3	0.6	10.8	0.9
Gallbladder wall	201	80.4	213	83.5	189	88
Left colon	16.6	1.9	15	0.5	18.2	0.9
Small Intestine	42.6	13.1	38.9	9.6	46.3	16.5
Stomach Wall	15.3	1.5	13.9	0.1	16.7	0.5
Right Colon	19	0.9	18.9	1.3	19.1	0.5
Rectum	13.6	1.7	12.1	0.3	15.2	0.9
Heart wall	27.8	7.8	24.3	3.3	31.3	9.9
Kidneys	22.3	3.7	19.3	1.7	25.3	2.1
Liver	64.3	18.4	62.6	14.6	66.1	23.9
Lungs	14.7	2.3	13.6	2.2	15.8	2.1
Ovaries					15.6	0.5
Pancreas	20.5	3.6	17.4	0.6	23.6	1.9
Prostate			11.7	0.3		
Salivary Glands	10.3	1.2	9.5	0.7	11.2	0.9
Red Marrow	19.5	3.5	16.7	2	22.4	1.7
Osteogenic Cells	15.1	1.5	13.8	1	16.3	0.5
Spleen	27.5	12.2	22.9	2.7	32.1	16.8
Testes			8.6	0.7		
Thymus	12.4	1.5	11.2	0.4	13.7	0.8
Thyroid	10.5	1.1	9.7	0.6	11.3	0.8
Urinary Bladder Wall	22.9	7	21.7	7.8	24	7.1
Uterus	16.2	0.8				
Total body	13.2	2	11.3	0.3	15.1	0.2
Effective dose	17.5	2.1	15.7	0.5	19.4	0.8

**Table 8:** Radiation dosimetry (μSv/MBq) across fasting condition

Site	Non-fasting	SD	Fasting participants	SD	Partial-fasting participants	SD
	participants		mean		mean	
	mean					
Adrenals	23.6	2.2	22.6	3.6	25.5	2.8
Brain	13.1	1.4	16.3	4.3	14.8	3.3
Breasts						
Esophagus	14.3	1.8	14.2	1.5	14.8	2.2
Eyes	9.2	1.6	10.1	1.6	8.6	1.6
Gallbladder wall	155.5	2.1	270.5	33.9	107.6	30.3
Left colon	16.5	1.1	16.9	2.4	16.1	2.3
Small Intestine	45.3	8.8	34.6	4	55.9	21.4
Stomach Wall	15.2	1.8	15.4	1.7	15.3	1.9
Right Colon	18.3	0.4	19.7	0.5	18.2	1
Rectum	14	2.3	13.7	1.9	13.2	2
Heart Wall	21.5	3.1	30.8	9.9	28.1	0.9
Kidneys	21.2	4.5	22.5	3.6	23	5.7
Liver	68.5	3.5	50	8.2	88.8	10.3
Lungs	17.1	2.5	13.9	2.1	13.9	1.6
Ovaries						
Pancreas	20.7	3.8	19.7	3.4	21.8	5.8
Prostate						
Salivary glands	10.1	1.3	11	1.1	9.3	1
Red marrow	20.3	1	18.7	3.6	20.3	6.4
Osteogenic cells	15.5	0.2	15	1.6	14.9	2.8
Spleen	26	0.7	29.9	18.1	24.2	2.8
Testes						
Thymus	12.3	1.8	12.9	1.7	11.7	1.3
Thyroid	10.5	1.4	11	0.9	9.6	0.9
Urinary bladder wall	25.1	5.7	18.1	5.5	30.2	3.8
Uterus						
Total body	13.2	2.6	13.3	2	13.1	3
Effective dose	17.6	2.4	17.3	2.2	17.9	3

## Discussion

We have described the regional distribution of ^18^F-VAT binding in eight healthy human volunteers with no evidence of neurologic or psychiatric disease. No adverse reaction was observed during whole-body PET scans of ^18^F-VAT to acquire data for human dosimetry assessment. Our data in healthy humans identifies the gallbladder as the critical organ, with its radiation burden varying depending on fasting status. When averaged across all participants in various fasting conditions the overall radiation burden to the gallbladder wall was 201.0 µSv/MBq (502 mrem/mCi); the second highest radiation dose is delivered to the liver which received an average of 64.3 µSv/MBq (188 mrem/mCi). Without accounting for fasting state, our organ dosimetry thus indicate that suitable doses of ^18^F-VAT can safely be administered to obtain PET imaging data where the calculated effective dose was 15.7 and 19.4 µSv/MBq for males and females, respectively. Specifically, the radiation exposure from a single dose of 249 MBq (6.7 mCi) or less avoids exceeding a gallbladder radiation dose of 50 mSv (5 rem). When comparing fasting, partial fasting, and non-fasting states, post-injection greater than pre-procedural oral caloric supplementation increases gallbladder clearance, thus reducing gallbladder wall radiation burden and allowing a higher administered dose to 465 MBq (12.6 mCi) without exceeding a gallbladder dose of 50 mSv (5 rem). These data indicate that oral caloric supplementation approximately 90 min after ^18^F-VAT injection can reduce the gallbladder wall radiation exposure, i.e., the critical organ, which is particularly important when higher radiotracer doses or multiple PET studies are desired in the same individual.

The observation of hepatobiliary clearance is consistent with a prior fasting non-human primate dosimetry study demonstrating that the critical organ is the liver [15]. This human cohort represents a 6.5-fold greater gallbladder wall radiation dose and longer residence time compared to that previously estimated from two non-human primates (mean 0.100 ± 0.048 h in humans versus 0.01 ± 0.0008 h in non-human primates). Despite this difference in gallbladder wall dosimetry, the liver dosimetry and residence time remained nearly equivalent across species. Discrepancies in the gallbladder dosimetry values across species may reflect variable hepatobiliary clearance rates secondary to dietary features, especially since all nonhuman primates were strictly fasted overnight and throughout 6 h PET procedures. Overall dietary lipid content, relative fasting state, etc., [24], or anesthesia may also influence hepatobiliary excretion of the radioligand in NHPs. Our observations that gallbladder radioactivity accumulation was reduced when ^18^F-VAT was given to non-fasting subjects, as well as by as much as 59% in following inter-PET segment oral caloric supplementation supports the conclusion that non-fasting state reduces the overall gallbladder wall residence time and radiation burden.

The effective dose (E) for ^18^F-VAT (averaged across genders) is 17.5 µSv/MBq (65 mrem/mCi), which does not appreciably limit ^18^F-VAT doses when performing PET studies of human participants. Injection of the maximum partial-fasting 465 MBq (12.6 mCi) dose of ^18^F-VAT would deliver a whole-body dose of only 8.1 mSv (0.81 mrem). This is well below the typical whole-body limit of 30 mSv (3 rem). The average E in these human studies and in prior PET studies of NHP was in good agreement (17.5 mSv/MBq and 14.1 mSv/MBq, respectively).

In the partial and non-fasters there was relatively elevated radiation dose to the small intestines, supporting the hypothesis that a normal physiologic response to oral fat intake, namely increased ejection of stored bile in the gallbladder is the mechanism for our findings. Together, our observations suggest that ^18^F-VAT clears through the hepatobiliary system at variable rates based on fasting state, and that oral ingestion following radiotracer injection reduces overall radiation dose to the gallbladder wall. These data suggest that ^18^F-VAT PET studies should be performed with oral supplementation following injection of the tracer (e.g., 20 ml of high-fat emulsion following ^18^F-VAT injection [25]) to reduce relative radioactive dose to the critical organ. While our data demonstrates a significant effect following oral supplementation 90 min after ^18^F-VAT injection, our study was not designed to identify the optimal post-injection timing of oral supplementation in reducing critical organ radiation.

Additional data in male Sprague Dawley Rats support the notion that the hepatobiliary system represents the highest ^18^F-VAT regional uptake [12]. However, since rats do not have a gallbladder [26] biodistribution studies in rats can be misleading for radiotracers in which the gallbladder is the critical organ in humans. An additional species differences relates to reduced relative renal ^18^F-VAT uptake in humans and non-human primates compared to rats. Thus, dosimetry studies conducted solely in rats remain inadequate when attempting to identify doses that comply with regulations for radiation exposure in humans.

The tissue dose from a radiotracer relates to both the radionuclide’s physical half-life (approximately 110 min for ^18^F) and biological half-life of the parent and radio-metabolites. Thus, organ radiation burden relates to the initial organ perfusion, retention until clearance and exposure from nearby organs. Highly perfused organs such as heart, lung, liver, kidney, and brain receive a larger fraction of the absorbed radiation and most radiotracer molecules undergo metabolism and are cleared by either the hepatobiliary (liver, gallbladder) or renal (kidneys, urinary bladder) systems. Similar to prior studies in the rat and non-human primate, we observe rapid renal uptake and clearance followed by cumulative urinary bladder radiation accumulation suggesting some urinary clearance [14]. Higher uptake in liver and gallbladder wall in non-human primates and humans suggests primary hepatobiliary clearance of ^18^F-VAT. This is not surprising since the hepatobiliary route is a common route of excretion for lipophilic compounds, supporting that administration of ^18^F-VAT to non-fasting participants may significantly reduce the critical dose burden to the gallbladder wall compared to fasting states, beyond urinary voiding between imaging segments that would reduce the absorbed dose to the urinary bladder.

## Conclusion

Our data demonstrate that administration of approximately 249 MBq (6.7 mCi) ^18^F-VAT would produce a total human effective dose of < 4.4 mSv in the fasting state. Oral intake approximately 90 min following injection will limit radiation dose to the gallbladder wall, the critical organ where up to 465 MBq (12.6 mCi) may be injected to produce a similar total human effective dose of < 8.1 mSv in the non-fasting state. Our human dosimetry study indicates that ^18^F-VAT is a safe and promising VAChT radioligand, serving as an effective imaging tool for assessing cholinergic deficit and monitoring therapeutic effects in neurological and psychiatric disease. Administering oral caloric supplementation after ^18^F-VAT injection can reduce critical organ radiation exposure when higher radiotracer doses or multiple PET studies are desired in the same individual. Moreover, our method of reducing gallbladder dose through dietary means may have broad application to other brain PET radiopharmaceuticals, since CNS drugs are typically lipophilic in order to cross the blood–brain-barrier, and hence would also be expected to be excreted by the same hepatobiliary route followed by ^18^F-VAT.

## Data Availability

De-identified data are available upon request to qualified investigators.
